# NeMo: Network Module identification in Cytoscape

**DOI:** 10.1186/1471-2105-11-S1-S61

**Published:** 2010-01-18

**Authors:** Corban G Rivera, Rachit Vakil, Joel S Bader

**Affiliations:** 1 Department of Biomedical Engineering and High-Throughput Biology Center, Johns Hopkins School of Medicine, Baltimore, MD 21218, USA

## Abstract

**Background:**

As the size of the known human interactome grows, biologists increasingly rely on computational tools to identify patterns that represent protein complexes and pathways. Previous studies have shown that densely connected network components frequently correspond to community structure and functionally related modules. In this work, we present a novel method to identify densely connected and bipartite network modules based on a log odds score for shared neighbours.

**Results:**

To evaluate the performance of our method (NeMo), we compare it to other widely used tools for community detection including kMetis, MCODE, and spectral clustering. We test these methods on a collection of synthetically constructed networks and the set of MIPS human complexes. We apply our method to the CXC chemokine pathway and find a high scoring functional module of 12 disconnected phospholipase isoforms.

**Conclusion:**

We present a novel method that combines a unique neighbour-sharing score with hierarchical agglomerative clustering to identify diverse network communities. The approach is unique in that we identify both dense network and dense bipartite network structures in a single approach. Our results suggest that the performance of NeMo is better than or competitive with leading approaches on both real and synthetic datasets. We minimize model complexity and generalization error in the Bayesian spirit by integrating out nuisance parameters. An implementation of our method is freely available for download as a plugin to Cytoscape through our website and through Cytoscape itself.

## Background

Rapid innovation in the field of high-throughput whole-genome biochemistry has revolutionized our understanding of biology. The vast amount of molecular biology data presents us with new organizational challenges as we seek to extract knowledge from whole-genome experimental assays. Biochemical assays that annotate associations between pairs of genes and proteins have become increasingly diverse. Some of these important assays yield protein-protein, protein-DNA, and synthetic lethal genetic interactions. Taken together these molecular interaction data sets form our picture of the known interactome. With estimates on the size of the complete protein interactome for humans and other metazoans topping 650,000 interactions [1-4], sophisticated tools are needed to cope with the complexity of biological systems.

In molecular interaction networks, groups of densely connected molecules frequently have an important biological interpretation. Dense sub-networks of molecules often represent protein complexes and coherent biological processes. Community finding in large networks has become a ubiquitous problem, and many research groups spanning diverse fields have proposed methods. Theoretically, the problem of finding the densest subgraph in a network is known to be NP-hard [5]. Consequently, almost all methods that propose solutions to this problem are necessarily approximate heuristics. In our preliminary analysis on synthetic and real datasets, we find our approach to be competitive with or significantly better than a selection of leading approaches including Metis, MCODE, and spectral clustering. Our method is also fast.

Dense subgraphs and clique structures are not the only interconnectivity pattern with biological significance. Bipartite structures in datasets of synthetically lethal interactions can represent redundancy in biological pathways. Our approach simultaneously identifies both dense network structures and dense bipartite structures in molecular interaction networks. Molecular interactions can be either directed in the case of transcriptional regulatory interactions or undirected in the case of protein-protein interactions. Our proposed technique allows both directed and undirected molecular interactions to be naturally integrated and processed using our approach.

Cytoscape [6] has proven to be a superior platform for biological network visualization and analysis. The integration of our network analysis tool with Cytoscape ensures broad dissemination and increased usability of our technique. Coupled with other network analysis tools that provide functional enrichment and topology statistics for subnetworks of interactions, we enable seamless integration with existing network analysis workflows.

### Previous research

The problem of identifying community structure has been studied by many fields including high performance computing, bioinformatics, applied mathematics, and soft matter physics. The general problem is known by several names including community detection, network module prediction, network clustering, and graph partitioning [7-9]. Existing approaches have limitations that we address with our method.

Spectral methods take advantage of the Fiedler vector of the graph Laplacian to perform recursive bisection and multiway partitioning [10,11]. Some spectral methods can be used to identify dense bipartite structures [12,13]. Spectral methods have associations with repeated random walks which can also be used to identify dense network modules [14,15].

Methods that identify minimum cuts or maximum flows are also used for network bisection [16,17]. Some of these methods aim to maximize a measure of modularity [18], although the measure of modularity is known to have resolution limits [19]. These approaches can also be applied to directed networks [20]. Heat kernels [21] and betweenness centrality [22] have also been used to identify community structure.

Other methods formulate a score to identify hubs or seed nodes and perform a local search to identify the community surrounding the hub [23-26] based on diverse fitness functions like mutual information. These methods can be fast for small queries, but frequently lack global properties. Methods that use bottom-up hierarchical agglomeration to identify community structure [27] are also frequently used.

MCODE [24] was introduced for the Cytoscape platform to enable searches for dense clique-like structures within a network. The algorithm identifies seed nodes for expansion by computing a score of local density for each node in the graph. The algorithm expands highly scoring seed nodes in a local search procedure by adding highly scoring nodes connected to the module. The algorithm includes post-processing features that remove unwanted elements from the set of resulting networks. The algorithm relies on many adjustable parameters, which can burden a user and possibly lead to over-fitting.

Spirin and Mirny [28] use a brute force bottom-up approach to enumerate all fully-connected graphs in the network. The approach has exponential time complexity and is not viable for large networks such as the molecular interaction networks observed today. They propose a Monte Carlo (MC) procedure to identify dense subnetworks as an optimization problem with network density as the objective. They use the simulated annealing algorithm to ensure convergence, although convergence may be slow for large networks. Others have suggested efficient methods for identifying cliques of a given size [29].

More recent approaches like that of Dhillon and Guan [30] perform graph-partitioning using weighted kernel k-means. The approach allows graph-partitions of unequal sizes. However, this and other top-down partitioning and bisection methods require the specification of the number of clusters. In networks of sufficient size and complexity, it is unreasonable to expect a user to know this value *a priori*.

Zhang *et al. *[31] find that many real world modules are not densely interconnected, which breaks a widely held assumption about the clique-like nature of network modularity. Indeed, many families of protein ligands do not interact within the family; however, these families can be identified by virtue of interactions with many of the same receptors. Thus, some modules form dense bipartite structures with other parts of the network. These details indicate that network modularity comes from both clique-like and dense bipartite network topologies. The method described here is unique in that it identifies both types of network communities in a single approach.

### Contributions of our approach

In this paper, we propose a novel network clustering approach called NeMo. Our results on both synthetic and real data indicate that NeMo has performance which is competitive with or better than a selection of widely used approaches. Our method identifies functional modules that are overlooked by many existing module finding algorithms, including dense bipartite graph structures. Additionally, our method can integrate diverse data sources such as undirected protein-protein interactions and directed protein-DNA interactions. Our method is more accessible to new users because there are no parameters to tune.

## Results

### Algorithm comparison on synthetic data

To verify the effectiveness of our approach, we compare two variants of the NeMo algorithm to a selection of widely used community finding algorithms including kMetis, MCODE, and spectral clustering. All of the methods have run time requirements small enough for interactive use. Metis is a fast and widely used as a benchmark for community finding algorithms. Karypis and Kumar [7], the authors of metis, indicate that kMetis produces more accurate clusters than metis. Thus, kMetis is used as the benchmark. The number of embedded clusters in each synthetic network varies from 5 to 10. kMetis has a parameter to specify the number of clusters. We run kMetis six times for each synthetic network to allow for partitions based on different numbers of clusters. Spectral clustering requires the number of bisections to be set. In the synthetic trials, we run spectral clustering with 2, 3 and 4 levels of recursive bisection. MCODE has a parameter that specifies the size of clusters returned. For each synthetic network, we run MCODE 10 times in a uniform grid search over the size parameter. All other MCODE parameters are left as defaults. For kMetis, MCODE, and spectral clustering, the set of putative modules for a synthetic network is taken to be the union of all modules for all parameter settings previously discussed. NeMo in contrast has no tuneable parameters.

NeMo uses hierarchical agglomerative clustering as part of the procedure. To identify the best setting for the hierarchical clustering, we compare the use of single-linkage and complete-linkage hierarchical agglomerative clustering as part of NeMo for synthetic network module identification.

The reconstruction fidelity of the synthetic network for an algorithm is given by the putative module of highest similarity to a synthetic module. We assign similarity between networks to be the Jaccard coefficient between the set of nodes in the synthetic module and the putative module. It should be noted that algorithms that return more putative modules benefit from this approach. Reconstruction fidelity is a measure that ranges from 0, completely missed, to 1, recovered exactly. We define reconstruction error as one minus reconstruction fidelity.

**Figure 1:**
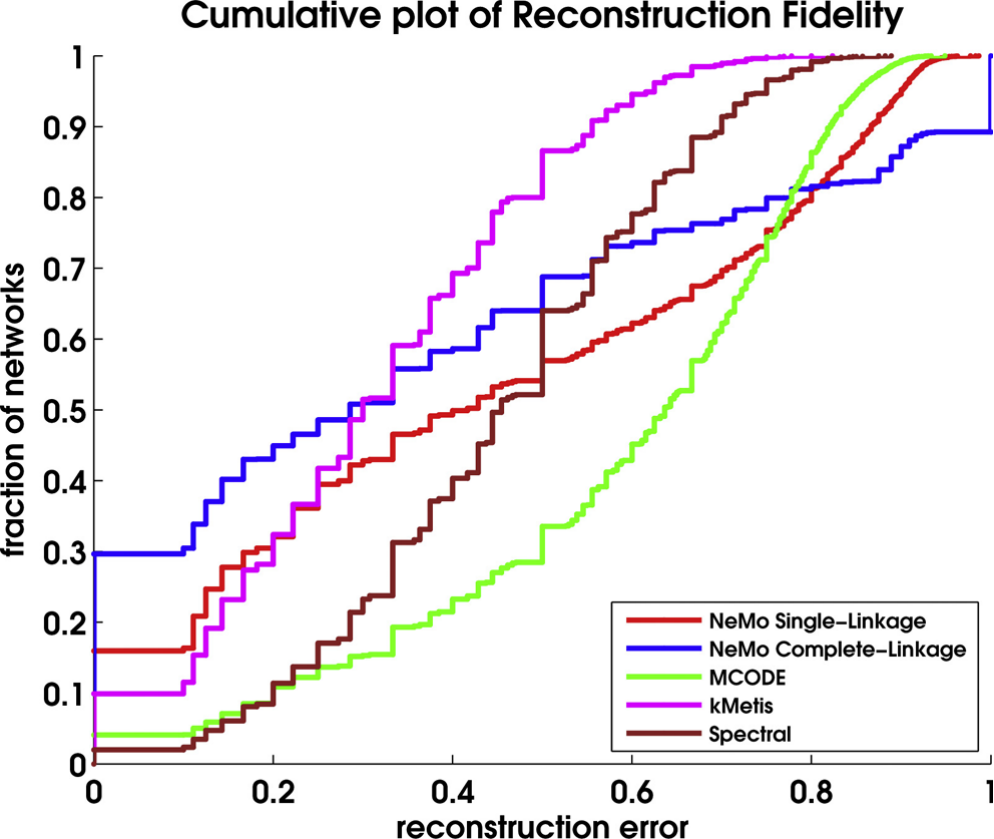
**Community-finding algorithm performance on synthetic networks**. Comparison of NeMo with single-linkage, complete-linkage, MCODE, kMetis, and spectral clustering measured in terms of the reconstruction fidelity of synthetic modules. The x-axis indicates reconstruction error between 0 and 1 with 0 indicating complete module reconstruction and 1 indicating that the algorithm did not identify the module. The figure shows the fraction of modules identified with a reconstruction error less than a given threshold.

In Figure 1, we show the result of the algorithm comparison on synthetic data. The dataset consisted of 1000 synthetic networks containing over 8000 embedded modules. The plot indicates that NeMo with complete-linkage hierarchical clustering identifies 30% of modules with 100% fidelity and 45% of modules with 80% fidelity. The results suggest that NeMo with complete linkage identifies modules with higher reconstruction fidelity than NeMo with single-linkage or MCODE. For reconstruction error less than 0.3, NeMo with complete-linkage performs better than kMetis, MCODE and spectral clustering. NeMo with single-linkage performs competitively with kMetis and better than MCODE and spectral clustering for reconstruction error levels less than 0.2.

The mean number of putative modules returned by each algorithm varied significantly. NeMo returned an average of 24 and 19 putative modules for single-linkage and complete-linkage respectively, while MCODE returned an average of 426 putative modules for synthetic networks. kMetis returned 45 and spectral clustering returned 22 on average.

### Algorithm comparison using MIPS human complexes

To remove potential bias that may be introduced by using synthetic data, we compare the algorithms based on the entire human interactome. We use the complete set of MIPS human complexes [32,33] as the gold standard set of network modules. We generate putative network modules from the entire human interactome using each algorithm. The human interactome used in this analysis is a comprehensive set of more than 225,000 physical human protein-protein interactions (PPIs) taken from the Michigan Molecular Interactions (MiMI) [34] repository. MiMI aggregates physical PPIs from many reputable sources including REACTOME, DIP, BIND, HPRD, and others.

Motivated by the notion that the definition of module reconstruction fidelity does not control for the size of the putative module set, we present the algorithm comparison using a new procedure. For example, an algorithm that returns all combinations of nodes as putative network modules would have high reconstruction fidelity for all test modules. To control for the size of the putative module set, we use the algorithms to predict if a given network module is a real MIPS complex or a randomly generated network. The score used for prediction is the measure of reconstruction fidelity. Under the new test, an algorithm that returns all combinations of nodes as putative network modules would appear no better than random.

The dataset consists of a gold standard set of 380 human MIPS complexes. We generate 380 randomized complexes by permuting node labels. The randomized complexes preserve the size distribution of the MIPS complex dataset. For each algorithm, we compute the set of putative network modules embedded in the interactome. We use the putative network module set generated by each algorithm to rank all 760 test modules by reconstruction fidelity. To display the results, we generate a receiver operating characteristic (ROC) curve. The ROC characterizes the true positive rate and false positive rate for MIPS complex prediction for varying levels of reconstruction fidelity. The best methods will have high reconstruction fidelity for real MIPS complexes and low reconstruction fidelity for random complexes.

**Figure 2:**
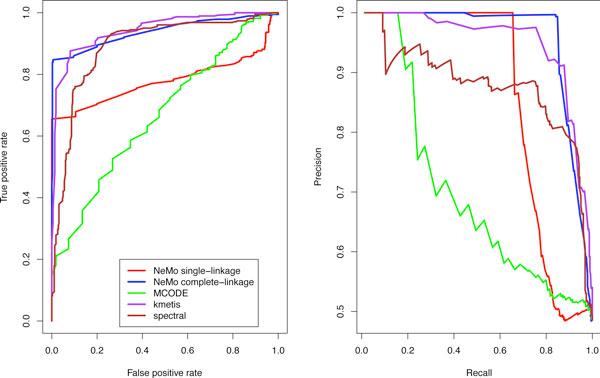
**Interactome-scale community-finding algorithm comparison**. (a) ROC comparing NeMo with single-link, NeMo with complete-linkage, MCODE, kMetis, and spectral clustering for the identification of MIPS human complexes. (b) Precision and recall curves comparing NeMo with single-link, NeMo with complete-linkage, MCODE, kMetis, and spectral clustering for the identification of MIPS human complexes. Each algorithm produced a set of putative network modules embedded in the interactome. The putative network module set of each algorithm was used to rank a set of 380 MIPS complexes and 380 randomized networks by reconstruction fidelity (Jaccard coefficient).

From the ROC curve (Figure 2(a)) we find that, for a false positive rate less than 0.1, NeMo with complete-linkage has a better true positive rate than kMetis, MCODE, and spectral clustering. With a false positive rate of zero, we find that NeMo with complete-linkage has the highest true positive rate of about .85. We computed the area under the curve for each algorithm, and we find that NeMo with complete-linkage has an AUC of 0.94, kMetis has an AUC of 0.94, spectral clustering has 0.89, NeMo with single-linkage has 0.77, and MCODE has 0.67. We conclude from this data that NeMo with complete-linkage has performance on real data that is competitive with kMetis and spectral clustering and better than MCODE.

The precision recall curve (Figure 2(b)) highlights another aspect of the comparison. NeMo with complete-linkage maintains 100% precision from 0% to 85% recall, while spectral clustering has a significant drop to 90% precision with only 10% recall.

All methods experience a dramatic drop in precision at 85% recall. Beyond this threshold, the methods can no longer distinguish real MIPS complexes from random modules.

### Application to the CXC chemokine pathway

The CXC chemokine pathway is important in regulating inflammation response. The inflammation response is known to be an important inhibitor of cancer progression. To study the relationship between cancer and the CXC chemokine pathway, we examined the CXC chemokine pathway with respect to high-grade glioblastoma [35].

**Figure 3:**
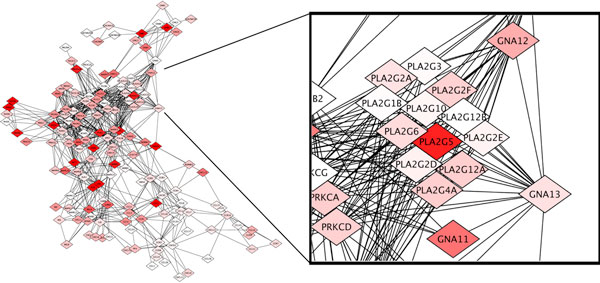
**A functional module of the CXC chemokine pathway uniquely identified by NeMo**. A collection of 12 proteins from the glycine, serine and threonine metabolic pathway. The family of proteins can be identified visually, but is often missed by automatic network module identification algorithms because they form an independent set in the CXC chemokine pathway.

We used NeMo to identify functionally related modules in the network. For illustrative purposes, in Figure 3, we highlight one functionally related module uniquely identified by our method. We identify the family of 12 glycine, serine and threonine metabolic proteins including PLA2G2E, PLA2G3, PLA2G2A, PLA2G2D, PLA2G4A, PLA2G12B, PLA2G2F, PLA2G5, PLA2G6, PLA2G12A, PLA2G10, and PLA2G1B. Accession numbers and gene names are given in Table 1. The functional module is missed by other network partitioning and clustering methods because the family of proteins does not self-interact. In fact, they form an independent set in the CXC chemokine pathway. In Figure 3, we show the family of phospholipases in the context of the CXC chemokine pathway. The intensity of red node coloring indicates elevated levels of gene expression in association with glioblastoma. We find that PLA2G5 experiences relatively high levels of gene expression in association with high-grade CNS glioblastoma.

**Table 1:** Genes discussed in association with the CXC chemokine pathway module

Symbol	Acc.	Full Name
PLA2G2E	Q9NZK7	phospholipase A2, group IIE
PLA2G3	Q9NZ20	phospholipase A2, group III
PLA2G2A	P14555	phospholipase A2, group IIA (platelets, synovial fluid)
PLA2G2D	Q9UNK4	phospholipase A2, group IID
PLA2G4A	P47712	phospholipase A2, group IVA (cytosolic, calcium-dependent)
PLA2G12B	Q9BX93	phospholipase A2, group XIIB
PLA2G2F	Q9BZM2	phospholipase A2, group IIF
PLA2G5	P39877	phospholipase A2, group V
PLA2G6	O60733	phospholipase A2, group VI (cytosolic, calcium-independent)
PLA2G12A	Q9BZM1	phospholipase A2, group XIIA
PLA2G10	O15496	phospholipase A2, group X
PLA2G1B	P04054	phospholipase A2, group IB (pancreas)
GNA12	Q03113	guanine nucleotide binding protein (G protein) alpha 12
GNA13	Q14344	guanine nucleotide binding protein (G protein), alpha 13

We also notice the high connectivity between GNA12 and GNA13 and the family of phospholipases. It is known that GNA12 and GNA13 are associated with long-term depression and regulation of actin cytoskeleton. The jointly up-regulated expression of both the phospholipases and GNA12 and GNA13 suggests a relationship between glioblastoma, inflammation, and depression. The relationship between inflammation and depression is well characterized [36]. While the relationships between inflammation, depression and cancer has been identified [37] there does not seem to be existing literature that suggests a relationship between glioblastoma, inflammation, and depression specifically.

### Integration into Cytoscape

To increase the accessibility and utility of our method in practice, we implemented our algorithm as a plugin to Cytoscape. Currently, the plugin can be downloaded from our website [38]. The plugin is distributed containing NeMo with complete-linkage.

## Discussion

### Identifying dense bipartite networks

All of the methods described in the related work section identify either dense networks or dense bipartite structures. The method we present here identifies both network topologies in a common approach. Because NeMo does not rely on direct interaction to identify community structure, the method can identify an interesting set of structures that lie between clique-like networks and dense bipartite graphs.

### Applicable to directed and undirected graphs

NeMo allows the integration of protein-protein, protein-DNA, and metabolic interaction networks to find densely-connected components. Our method seamlessly identifies functional modules in both directed and undirected graphs. Furthermore, our method is able to identify network modules in combined directed and undirected networks.

### Parameterless design

NeMo has no adjustable parameters. The lack of tunable parameters is an advantage for inexperienced users, and appears to boost performance as well. Methods such as kMetis, k-means, spectral clustering and many other top-down approaches require the user to select the number of clusters in advance. In a network of sufficient complexity and size, a user should not be expected to know the number of embedded network modules *a priori*. The number of clusters is one of many parameters used by existing methods. In these methods, an unfortunate choice of parameter setting can lead to a poor result set. NeMo avoids many of these complications by using the maximum likelihood estimates for the parameters in the method.

### Algorithm comparisons

To evaluate our proposed method, we compared NeMo with widely used community finding algorithms like kMetis, MCODE, and spectral clustering. We found that NeMo with single-linkage was outclassed by modern community finding approaches like kMetis and spectral clustering. We find that NeMo with complete-linkage has performance which is competitive with or better than recent approaches on both real and synthetic data. As a general trend in the results, we find that NeMo with complete-linkage identifies more networks with 100% reconstruction fidelity than the competing approaches; however, the advantage is lost for higher acceptable levels of false positives and reconstruction error.

## Conclusion

As the size of the known human interactome grows, biologists increasingly rely on computational tools to identify patterns in the data. In this work we present a novel community finding algorithm based on a log odds score of shared neighbours. NeMo is unique in its ability to identify both dense network and dense bipartite structures in a single approach. To evaluate the performance of our method (NeMo with complete-linkage), we compare our method to a set of widely used approaches for community finding such as kMetis, MCODE, and spectral clustering. We test all of the methods using a collection of synthetically constructed networks and the entire human interactome. On both real and synthetic datasets, we find that NeMo with complete-linkage has performance that is competitive with or better than existing methods for community finding.

We apply our method to the CXC chemokine pathway to identify functional modules. We highlight a functional module of 12 disconnected phospholipase isoforms. The result reveals our methods ability to identify coherent functional modules that are weakly connected. We implemented NeMo with complete-linkage as a plugin for Cytoscape. The plugin is freely available through our website [38] and through Cytoscape itself.

## Methods

We propose a log odds score *r*_*ab *_for observing a certain number *s*_*ab *_of shared neighbours between nodes *a *and *b*. A shared neighbour is a node *c *that satisfies *a~c *and *b~c*, where the tilde symbol indicates adjacency. We assume that the counts *s*_*ab*_~Poisson(*λ*). The score *r*_*ab *_approximately equals the log odds ratio between the probability of *s*_*ab *_under the alternative and null hypotheses. The null hypothesis is that the number of shared neighbours between *a *and *b *is from a random network model. We define  as the Poisson parameter for *s*_*ab *_under the null hypothesis. The alternative hypothesis states that the number of shared neighbours between *a *and *b *is greater than expected by chance. We define  as the Poisson parameter for *s*_*ab *_under the alternative hypothesis.(1)

Simplifying equation (1), we have,(2)

If we assume *s*_*ab*_~Poisson(*λ*),(3)

Simplification of equation (3) gives,(4)

To find the maximum likelihood solution for , we solve,(5)

Solving equation (5), we find the maximum likelihood solution.(6)

Under the null hypothesis,  is the expectation of *s*_*ab*_. Let *n*_*a*_, *n*_*b*_, and *e *be the number of neighbours of *a*, the number of neighbours of *b*, and the total number of edges respectively. Let *N *be the set of all nodes.(7)

Substituting equation (6) in equation (4) gives the score(8)

Finally, we exclude significance from node pairs that have far fewer shared neighbours than expected at random.(9)

### The Grouping Process

We compute the score *r*_*ab *_for all node pairs *a *and *b*. We perform hierarchical agglomerative clustering using either single-linkage or complete-linkage clustering. We process node pairs in descending order based on the score *r*_*ab*_. The process ends when the observed number of shared neighbours for a node-pair is less than the expected number of shared neighbours. The interpretation of the convergence criteria is that we have processed all node pairs that have more shared neighbours then we would expect by chance.

We follow a simple procedure to collapse insignificant structure from the hierarchical tree. For every internal node *p *with two children *m *and *n *where *m *is a leaf and *n *is an internal node, we collapse the edge between *p *and *n*. Putative network modules are identified as the set of leaf nodes that are descendants of an internal node. A putative network module is returned for each internal node.

### Synthetic data construction

We aim to quantitatively evaluate the performance of our method with leading methods in the field. We use synthetically created networks to achieve this task. Each synthetic network consists of between 5 to 10 embedded clusters. The between cluster edge density is chosen uniformly at random between 0.05 and 0.1. Each cluster has an edge density chosen uniformly at random between 0.05 and 0.08. The size of each cluster is chosen randomly between 5 and 10.

## Competing interests

The authors declare that they have no competing interests.

## Authors' contributions

CGR and RV implemented the method, performed the analysis, generated the images and wrote the paper. JSB conceived of the algorithm and wrote the paper.
